# Discordance in Spirometric Interpretations Based on Korean and Non-Korean Reference Equations

**DOI:** 10.1186/2052-4374-25-42

**Published:** 2013-12-27

**Authors:** Nami Kim, Mi-Hee Park, Se-yeong Kim, Chunhui Suh, Sangyoon Lee, Kun-Hyung Kim, Chae-Kwan Lee, Dae-Hwan Kim, Jong-Tae Lee

**Affiliations:** 1 Department of Occupational and Environmental Medicine & Institute of Environmental and Occupational Medicine, Busan Paik Hospital, Inje University, 75, Bokji-ro, Busanjin-gu, Busan 633-165, Republic of Korea; 2 Occupational Medical Examination Center, Good Morning Hospital, 110, Samsan-ro, Nam-gu, Ulsan 680-804, Republic of Korea; 3 Department of Occupational and Environmental Medicine, Inje University Haeundae Paik Hospital, 875, Haeundae-ro, Haeundae-gu, Busan 612-896, Republic of Korea

**Keywords:** Spirometry, Spirometric reference equation, Workers, Punlmonary function test

## Abstract

**Objectives:**

Korean regression models for spirometric reference values are different from those of other ethnic groups. The purpose of this study was to evaluate discordance in spirometric interpretations based on using Korean reference equations versus non-Korean reference equations.

**Methods:**

Spirometry was performed on 825 Korean male workers from April 2009 to November 2011. The spirometric patterns and disease severity were evaluated using two Korean equations (Choi's and Lee's) and three equations for Caucasians (NHANES **III**, Morris's, and Knudson's), and the results of Choi's equation were compared with the non-Korean equations. The spirometric patterns were defined as normal, restrictive, and mild and moderate obstructive.

**Results:**

The mean differences in the FEV_1_% and FVC% between the two Korean equations were 2.0 ± 1.3% and 3.5 ± 2.2%, respectively. Morris's equation had the greatest difference in the FEV_1_% from Choi's equation: 32.9 ± 8.5%. Knudson's equation had the greatest difference in the FVC% from Choi's equation: 10.5 ± 6.5%.

**Conclusions:**

The two Korean equations shared similar characteristics of spirometric interpretation. The spirometric interpretations of Choi's equation were significantly different from those of Morris's equation and Knudson's equation.

## Introduction

Spirometry is one of the basic examinations in a pulmonary function test, and is widely applied for the periodic evaluation of workers who are exposed to pulmonary risk factors [1]. For spirometric interpretation, a normal reference value is necessary for determining the normal range, and this is calculated from spirometric reference equations. These reference equations should be established by performing measurements with standardized devices and methods. In addition, reference equations should be developed taking into account the specific age, height, sex, and ethnicity of tested subjects [2].

For the Korean general population, spirometric reference equations have been developed; thus, theoretically, these Korean spirometric reference equations should be applied to the Korean population [3]. For example, recently, a Korean spirometric reference equation based on the 2002 Korean National Health and Nutrition Examination Survey (KNHANES) was developed by Choi, using representative subjects for all adult age groups [4]. However, non-Korean spirometric reference equations published by Morris et al. in 1971 and Knudson et al. in 1983 have been widely used in Korea. These two reference equations were developed in a different environment from that of the current spirometric guidelines.

The 2011 American College of Occupational and Environmental Medicine (ACOEM) guideline recommends that the National Health and Nutrition Examination Survey (NHANES) III equation developed in 1999 be used [5]. Because Morris’s and Knudson’s equations, which were developed in 1971 and 1983, were not based on current spirometric guidelines [2], it has been reported that spirometric interpretations by these equations show differences from those by the NHANES III equation, which is comparatively newer [6,7].

It has also been reported that interpretations of spirometric data from a Korean population using non-Korean spirometric equations are different than in the population for which the equations were designed [4,8]. When a non-Korean spirometric reference equation based on a non-Korean population is applied in Korea, the spirometric interpretation could be in error due to ethnic differences.

According to studies outside Korea, the normal reference values of spirometry for ethnic Asian and African-American populations are thought to be lower than those for Caucasian populations [9-11]. However, the results of studies in Korea are different. Instead, Choi’s normal reference values were higher than those of Morris’s and Knudson’s in a Caucasian population [4]. In addition, the normal reference value developed by Lee et al. was estimated for the population aged 40 and over in two Korean cities, and it was also higher than that of Morris’s [8]. Moreover, in the estimation from Choi’s reference equation, the rate of decrease of spirometry by age in the Korean population was found to be lower than those of Morris’s and Knudson’s equations [4]. It was reported that Choi’s and Morris’s reference equations showed significant differences in application in a clinical setting [12,13].

Eom et al. evaluated spirometry for adults aged 40 and over in the cement factory areas of Chungcheongbuk province and concluded that the reference equation based on the NHANES III was more appropriate than that of Choi. However, this result is difficult to generalize to the whole Korean population because of the limited geographic area and age group of the sample [14]. Comparative studies of the reference equations of Choi and the NHANES III have been insufficient.

Therefore, in this study, the Korean reference equations of Choi et al [4]. and Lee et al [8]., which were developed based on groups aged 40 and above in a few cities of Gyeonggi province, were compared to verify the validity of Choi’s equation in new spirometry data we collected from workers of Pusan metropolitan city and other areas in Gyeongsangnam province. In addition, Choi’s equation was compared to three non-Korean equations - Morris’s, Knudson’s, and that of the NHANES III, and differences in the normal reference values for evaluating pulmonary dysfunction by each equation were investigated to find an ideal reference equation for accurate interpretation of Korean spirometric data.

## Materials and methods

### Subjects

The subjects in this study were 1,090 male workers who underwent spirometry in special occupational health examinations in 2 shipyards, a metal manufacturer, and their subcontractors located in Busan metropolitan city and other areas of Gyeongsangnam province from April 2009 to November 2011 [15].

Among them, non-ethnic Korean workers were excluded. The 825 subjects with satisfactory results with regard to suitability and reproducibility were selected for inclusion by analyzing the spirometry data with the Quality Control Guideline of Pneumoconiosis and Pulmonary Function Test of the Korea Occupational Safety and Health Agency (KOSHA) [16].

### Data collection

The subjects’ basic data such as age, height, and weight were collected by general physical examination. A chest X-ray was performed. The participants were asked about their smoking history and any breathing difficulties through a self-administered questionnaire before the spirometry.

For the spirometry, Forced Vital Capacity (FVC) and Forced Expiratory Volume in 1 second (FEV_1_) were measured. Then, to acquire values with percentages, the actual measured FEV_1_ and FVC were divided into the calculated FEV_1_ and FVC from each equation (FEV_1_% = measured FEV_1_/calculated FEV_1_, FVC% = measured FVC/calculated FVC). For the normal reference values, Choi’s and Lee’s reference equations were used as the Korean equations, while the NHANES III, Knudson’s, and Morris’s reference equations were applied as the non-Korean equations.

Age and height information was necessary in the calculation of all of the reference equations except Choi’s. For Choi’s equation, weight information as well as age and height information was needed. Lee’s equation was applied to those aged 40 and above only because that equation was developed based on a group aged 40 and above. The NHANES III has three types of reference equations for Caucasian, African-American, and Mexican-American populations. Among them, the Caucasian equation was used [4,8,10,17,18].

Comparing the measured values and normal reference values, pulmonary dysfunction classified into one of three patterns: normal, restrictive pulmonary disease, and obstructive pulmonary disease. The normal condition was defined as a value of the FVC of 80% and above and a value of the FEV_1_/FVC% of 70% and above the reference values. Restrictive pulmonary disease was evaluated as a value of the FVC of less than 80% of the reference values. Obstructive pulmonary disease was evaluated as values of the FEV_1_/FVC% of less than 70% of the reference values; mild obstructive pulmonary disease was defined as a value of the FEV_1_ of 80% and above, and moderate cases were defined as less than 80% of the reference value.

Spirometry was performed in subjects in stable condition after explaining its purpose and the required position. The spirometry was performed in a sitting position after one practice test. The better result of two attempts was selected. A FlowScreen testing device (Cardinal Health, Inc., Germany) was used for the screening.

Only the results considered reproducible and compliant with the pneumoconiosis guideline of the Korea Occupational Safety and Health Agency (KOSHA) were selected [16,19].

### Data analysis (Statistics)

The subjects’ ages were stratified into three groups: 20–39 years old, 40–49 years old, and 50 years and above. The differences in the values of the FEV_1_% and the FVC% between Choi’s and Lee’s equations were analyzed. The agreement of the evaluation for pulmonary dysfunction with the reference equations, and the concordance coefficient were analyzed.

The differences in the values of the FEV_1_% and the FVC% among Choi’s and the three non-Korean equations were analyzed. The agreement of the evaluation for pulmonary dysfunction with the reference equations and the concordance coefficients was analyzed.

A paired t-test was performed for the difference in the values of the FEV_1_% and the FVC%, and the concordance coefficient was analyzed by the Kappa distribution. PASW statistical software version 18.0 was used for the analysis.

## Results

### Subjects’ characteristics

The mean age of the 825 subjects was 44.5 ± 10.7. Of the subjects, 299 (36.2%) were distributed in the group aged 20–39, 232 (28.1%) were in the group aged 40–49, and 294 (35.6%) were in the group aged 50 and above. The average height was 169.9 ± 5.7 cm, and the mean weight was 68.3 ± 9.2 kg. Normal findings were shown in 732 subjects (88.7%) and abnormal findings were found in 93 (11.3%) on the chest X-ray. The number of smokers was 513 (62.2%). The number of non-smokers and past-smokers grouped together was 288 (34.9%). 121 subjects (14.7%) had obstructive pulmonary disease, which was defined as having a FEV_1_/FVC% of less than 70% of the reference value (Table 1).

**Table 1:** Characteristics of the study participants

	**n (%)**	**mean ± SD**
Age (years)		44.5 ± 10.7
20-39	299 (36.2)	
40-49	232 (28.1)	
≥ 50	294 (35.6)	
Smoking History		
Current smoker	513 (62.2)	15.8 ± 7.5*
Non-current smoker	288 (34.9)	
Height (cm)		169.9 ± 5.7
Weight (kg)		68.3 ± 9.2
Chest X-ray		
Normal	732 (88.7)	
Abnormal	93 (11.3)	
FEV_1_ (ℓ)		3.6 ± 0.6
Difference† (95% CI)		0.14 (0.05-0.23)
FVC (ℓ)		4.6 ± 0.7
Difference† (95% CI)		0.16 (0.06-0.26)
FEV_1_/FVC (%)		
≥70	704 (85.3)	
<70	121 (14.7)	

* cigarettes per day.

† difference between current smoker and non-current smoker.

### Comparing spirometric results using Choi’s and Lee’s reference equations

The result of the FEV_1_% by Choi’s equation was 2.0 ± 1.3% higher than that by Lee’s equation. The Kappa correspondence coefficient for the evaluation of obstructive pulmonary disease severity by the FEV_1_% was high, at 0.989. The FVC% by Choi’s equation was 3.5 ± 2.2% higher than that by Lee’s; specifically, it was 2.4 ± 1.9% higher in the group aged 40–49 and 4.5 ± 2.0% higher in the group aged 50 and above. In evaluating restrictive pulmonary disease by the FVC%, the concordance coefficient was 0.653, which shows a substantial agreement level. By age group, the concordance coefficient in the group aged 40–49 was 0.451, which means there was a moderate level of agreement, and in the group aged 50 and above, it was 0.728, which is a substantial agreement level (Tables 2 and 3).

**Table 2:** **Comparison of FEV**_**1**_**% and FVC% by the Korean equations**

	**Age group**	**Choi**	**Lee**	**Mean difference (95% CI)**
Measured/Calculated	40-49	93.6 ± 10.6	95.7 ± 10.8	-2.1 ± 1.1*
				(-2.3 - -2.0)
FEV_1_(%)	≥ 50	92.1 ± 11.9	92.8 ± 14.8	-1.9 ± 1.5*
				(-2.1 - -1.7)
	Total	92.1 ± 11.9	94.1 ± 13.3	-2.0 ± 1.3*
				(-2.1 - -1.9)
Measured/Calculated	40-49	98.9 ± 11.3	101.3 ± 11.4	-2.4 ± 1.9*
				(-2.6 - -2.1)
FVC(%)	≥ 50	97.6 ± 11.3	101.2 ± 12.8	-4.5 ± 2.0*
				(-4.7 - -4.2)
	Total	97.6 ± 11.3	101.2 ± 12.2	-3.5 ± 2.2*
				(-3.7 - -3.3)

* p-value < 0.01 by paired t-test.

**Table 3:** Concordance of spirometry results in the Korean equations

**Severity**	**Age**	**Choi**	**Lee**	**Agreement of Lee with Choi**	**Kappa**
Obstructive					
mild†	40-49	18 (7.8)	18 (7.8)	18 (100)	1.000*
moderate		3 (1.3)	3 (1.3)	3 (100)	
mild	≥ 50	48 (16.3)	50 (17.0)	48 (100)	0.985*
moderate		39 (13.3)	37 (12.6)	37 (94.9)	
mild	Total	66 (8.7)	68 (12.6)	66 (100)	0.989*
moderate		42 (5.9)	40 (7.4)	40 (95.2)	
Restrictive	40-49	10 (4.3)	3 (1.3)	3 (30.0)	0.451*
	≥ 50	22 (7.5)	13 (4.4)	13 (59.1)	0.728*
	Total	32 (4.8)	16 (3.0)	16 (50.0)	0.653*

Unit: No. of persons (%).

* p<0.01.

† Mild obstructive: FEV_1_/FVC (%) < 70 and FEV_1_% ≥ 80.

Moderate obstructive: FEV_1_/FVC (%) < 70 and FEV_1_% < 80.

Restrictive: FVC% < 80.

### Comparing spirometric results using Choi’s and the three non-Korean equations

With all three of the non-Korean equations, higher pulmonary vital capacities were interpreted based on the FEV_1_% and FVC% than with Choi’s equation. Among the three non-Korean equations, the values by the NHANES III equation were the closest to those by Choi’s; the value of the FEV_1_% was higher than Choi’s by 4.2 ± 2.5% and the value of the FVC% was higher than Choi’s by 1.5 ± 4.1%. The value of the FEV_1_% by Morris’s equation showed the biggest difference from that by Choi’s; the value of the FEV_1_% by Morris’s equation was higher by 32.9 ± 8.5% than that by Choi’s. In evaluating the severity of obstructive pulmonary disease, the Kappa concordance coefficient of both equations was 0.804. The value of the FEV% by Knudson’s equation showed the biggest difference from that by Choi’s; the value of the FEV% by Knudson’s equation was higher by 10.5 ± 6.5% than that by Choi’s. In evaluating the severity of obstructive pulmonary disease, the Kappa concordance coefficient of both equations was 0.119 (Tables 4, 5, and 6).

**Table 4:** **Comparison of FEV**_**1**_**% and FVC% of the non-Korean equations with Choi's equation by age group**

	**Age group**	**Choi**	**NHANES III**		**Knudson**		**Morris**	
		**Value**	**Value**	**Difference from Choi (95% CI)**	**Value**	**Difference from Choi (95% CI)**	**Value**	**Difference from Choi (95% CI)**
FEV_1_%	20-39	97.5 ± 10.0	94.0 ± 10.0	-1.8 ± 1.0†	97.6 ± 10.6	-5.3 ± 3.7†	117.5 ± 13.0	-25.2 ± 4.1†
				(-1.9 - -1.6)		(-5.7 - -4.9)		(-25.7 - -24.8)
	40-49	98.9 ± 11.3	97.8 ± 11.2	-4.3 ± 1.0†	104.7 ± 13.2	-11.1 ± 4.6†	126.9 ± 14.5	-33.3 ± 4.2†
				(-4.4 - -4.1)		(-11.7 - -10.5)		(-33.8 - -32.7)
	≥ 50	97.6 ± 11.3	97.5 ± 15.5	-6.6 ± 1.7†	106.3 ± 17.6	-15.4 ± 6.1†	131.4 ± 21.2	-40.5 ± 7.4†
				(-6.8 - -6.4)		(-16.1 - -14.7)		(-41.4 - -39.7)
	Total	97.6 ± 11.3	96.3 ± 12.7	-4.2 ± 2.5†	102.7 ± 11.8	-10.5 ± 6.5†	125.1 ± 17.8	-32.9 ± 8.5†
				(-4.4 - -4.0)		(-11.0 - -10.1)		(-33.5 - -32.4)
FVC%	20-39	97.5 ± 10.0	95.3 ± 9.6	2.1 ± 2.1†	101.4 ± 10.4	-3.9 ± 4.3†	97.1 ± 10.0	0.4 ± 2.5*
				(1.9 - 2.3)		(-4.4 - -3.4)		(0.1 - 0.6)
	40-49	98.9 ± 11.3	99.6 ± 11.3	-0.8 ± 1.8†	111.0 ± 13.5	-12.1 ± 5.2†	102.5 ± 11.6	-3.6 ± 1.9†
				(-1.0 - -0.5)		(-12.8 - -11.4)		(-3.9 - -3.4)
	≥ 50	97.6 ± 11.3	102.3 ± 13.0	-5.6 ± 3.2†	118.1 ± 16.2	-21.4 ± 8.2†	104.7 ± 13.2	-8.1 ± 2.6†
				(-6.0 - -5.3)		(-22.4 - -20.5)		(-8.3 - -7.8)
	Total	97.6 ± 11.3	99.0 ± 11.7	-1.5 ± 4.1†	110.1 ± 15.3	-12.5 ± 9.7†	101.4 ± 12.1	-3.8 ± 4.3†
				(-1.7 - -1.2)		(-13.1 - -11.8)		(-4.1 - -3.5)

* p-value < 0.05, † p-value < 0.01 by paired t-test.

**Table 5:** Concordance coefficient of obstructive lung staging in non-Korean equations and in Choi's equation

**Ages**	**Obstructive lung disease staging**¶	**Choi**	**NHANES III**		**Knudson**		**Morris**	
		**n (%)**	**n (%)**	**Agreement with Choi**	**n (%)**	**Agreement with Choi**	**n (%)**	**Agreement with Choi**
20-39	mild	6 (2.0)	7 (2.3)	6 (100)	8 (2.7)	6 (100)	12 (4.0)	6 (100)
	moderate	7 (2.3)	6 (2.0)	6 (85.7)	5 (1.7)	5 (71.4)	1 (0.3)	1 (14.3)
	Kappa			0.960†		0.920†		0.762†
40-49	mild	18 (7.8)	18 (7.8)	18 (100)	20 (8.6)	18 (100)	21 (9.1)	18 (100)
	moderate	3 (1.3)	3 (1.3)	3 (100)	1 (0.4)	1 (33.3)	0 (0.0)	0 (0.0)
	Kappa			1.000†		0.948†		-*
≥ 50	mild	48 (16.3)	60 (20.4)	48 (100)	67 (22.8)	48 (100)	81 (27.6)	48 (100)
	moderate	39 (13.3)	27 (9.2)	27 (69.2)	20 (6.8)	20 (51.3)	6 (2.0)	6 (15.4)
	Kappa			0.911†		0.859†		0.754†
Total	mild	72 (8.7)	85 (10.3)	72 (100)	95 (11.5)	72 (100)	114 (13.8)	72 (100)
	moderate	49 (5.9)	36 (4.4)	36 (73.5)	26 (3.2)	26 (53.1)	7 (0.8)	7 (14.3)
	Kappa			0.939†		0.893†		0.804†

Agreement rate% = by non-Korean equation / by Choi's Equation.

* Kappa not available.

† p-value < 0.01.

¶Mildly obstructive: FEV_1_/FVC (%) < 70 and FEV_1_% ≥ 80.

Moderately obstructive: FEV_1_/FVC (%) < 70 and FEV_1_% < 80.

**Table 6:** **Concordance coefficient of restrictive lung disease**^**† **^**in non-Korean equations and Choi's equation**

**Ages**	**Choi**	**NHANES III**		**Knudson**		**Morris**	
	**n (%)**	**n (%)**	**Agreement with Choi**	**n (%)**	**Agreement with Choi**	**n (%)**	**Agreement with Choi**
20-39	8 (2.7)	10 (3.3)	7 (87.5)	3 (1.0)	1 (12.5)	8 (2.7)	6 (75.0)
			0.771*		0.170*		0.743*
40-49	10 (4.3)	9 (3.9)	7 (70.0)	1 (0.4)	0 (0.0)	3 (1.3)	2 (20.0)
			0.726*		-0.008		0.294*
50-	22 (7.5)	8 (2.7)	8 (36.4)	2 (0.7)	2 (9.1)	5 (1.7)	5 (22.7)
			0.514*		0.156*		0.352*
Total	40 (4.8)	27 (3.3)	22 (55.0)	6 (0.7)	3 (7.5)	16 (1.9)	13 (32.5)
			0.643*		0.119*		0.449*

Agreement n (%) = n (by non-Korean equation / by Choi's equation *100).

* p-value < 0.01.

†Restrictive: FVC% < 80.

## Discussion

The equation of Choi et al [4]. was developed based on the KNHANES, which represents all age groups nationwide in Korea; however, the sample of the 206 male subjects finally included in Choi’s work was limited to representing the whole population of Korean males. In addition, unlike previous comparison studies, the estimated pulmonary vital capacity (the normal reference value) by Choi’s equation was found to be higher than that by the Caucasian equations in the case of the same age and height conditions, which means that the guideline for the normal reference value used here may be stricter than that used by the other studies [4].

In a comparison of the results by Choi’s and Lee’s equations to verify the validity of Choi’s equation, it was found that the results by these two equations were similar. Specifically, the difference in the FEV_1_ was only 2.0 ± 1.3%, and the concordance coefficient for the evaluation of the obstructive pulmonary disease severity was very high, at 0.989. The difference in the FVC% was 3.5 ± 2.2%, and the concordance coefficient for the evaluation of the restrictive pulmonary disease severity was relatively high, at 0.653. In a comparison of the normal reference values by the NHANES III and by the lung study of the Multi-Ethnic Study of Atherosclerosis (MESA) in healthy asymptomatic non-smokers out of 3,893 Americans, there were differences of -177 to 101 ㎖ in the FEV_1_ and -224 to 74 ㎖ in the FVC [20]; thus, a difference of about 2–3.5% may be acceptable. It was, therefore, concluded that Choi’s and the Lee’s equations support each other by sharing similar characteristics in spirometric interpretation.

The diagnosis rate of abnormal pulmonary vital capacity was higher by Choi’s equation than that by the other equations. A spirometric reference equation is for the estimation of abnormal pulmonary vital capacity instead of the confirmation of it; thus, it is expected to estimate 95% of test subjects as normal and 5% as abnormal out of the healthy general population. Although the population is in a comparatively healthy state, an extremely low rate for an abnormal group would indicate an unreliable validity for an equation. With Choi’s equation, the percentage evaluated as having restrictive pulmonary disease was estimated to be 4.8% out of the total subjects. The reference equation is used to evaluate the severity of obstructive pulmonary disease. The percentage of moderate obstructive pulmonary disease was estimated to be 5.9% by Choi’s equation, which means it was slightly higher than 5% of the total subjects. The percentage by the NHANES III, which showed the highest percentage among the three non-Korean equations was only 4.4%, which was thus below 5%. It is expected that the prevalence of obstructive pulmonary disease in the tested subjects would be higher than that of the general population because the subjects included workers who had been exposed to respiratory risk factors and smokers. However, with the NHANES III equation, the rate for moderate obstructive pulmonary disease was below 5%, which is the average expectation rate in a healthy population. In addition, it was far below 7.4%, the rate of moderate obstructive pulmonary disease evaluated in the American general population of all male subjects including smokers and those with respiratory disorders by the NHANES III equation [20].

Unlike the other equations, which used age and height information only as variables, it is worth noting that weight information was included in Choi’s equation. The average Korean height had risen dramatically from 164.9-167.7 cm in 1975 to 172.4-174.3 cm in 2010 for 17-year-old males [21]. There is reason to believe that weight information was included in Choi’s equation to reflect this trend in the Korean physique [4].

In comparing Choi’s equation with the non-Korean equations, there were two outstanding differences.

First, the evaluated subjects’ pulmonary vital capacity was higher by the non-Korean equations than that by Choi’s equation. Although the results were different with each equation, the three non-Korean equations tended to evaluate the FEV_1_% and the FVC% as higher values. Compared with Choi’s equation, the FEV_1_%, which is used to evaluate obstructive pulmonary disease, was particularly excessive, at 33.9%, by Morris’s equation, which is used the most in Korea; thus, moderate obstructive pulmonary disease was only estimated in 0.8% of the total subjects. The FVC%, which is used to evaluate restrictive pulmonary disease, was especially excessive, at 12.5%, by Knudson’s equation; thus, only 0.7% out of the total subjects were estimated to have restrictive pulmonary disease. Therefore, applying Morris’s and Knudson’s equations would raise an issue with their validity for a spirometric screening test. The concordance coefficient of the non-Korean equations and Choi’s equation are not low, but it is worth considering the clinical difference between the concordance coefficient and the actual diagnosis rate.

The over-evaluation level by the NHANES III was not as severe. The FEV_1_% and the FVC% by the NHANES III had the closest values to those by Choi’s equation. A comparatively higher concordance coefficient was found for the NHANES III evaluation than the concordance coefficients by the other equations. Although Morris’s and Knudson’s equations were certainly developed based on standardized devices and methods, they were developed in the past, which means these devices and methods have become out of date in the current setting. Thus, these equations have limited applicability today even if they are applied to the same ethnic populations. The NHANES III is the most recent equation; thus, it is comparatively free from this limitation. For this reason, the NHANES III equation could have been expected to show the most similar interpretation to the other equations in spite of the ethnic differences.

Second, the rate of decrease in the pulmonary vital capacity with aging was assumed to be lower with Choi’s equation than with the three non-Korean equations. This assumption also occurred in the process of developing Choi’s equation; the rate of decrease in the pulmonary vital capacity with increasing age was lower in Choi’s equation than in Morris’s and Knudson’s equations [4]. In comparing the NHANES III, this was confirmed as well in this study; the difference of the FEV_1_% between Choi’s and the NHANES III equations was only 1.8 ± 1.0% in the group aged 20–39; however, it was 6.6 ± 1.7% in the group aged 50 and above. The FVC% by the NHANES III was somewhat higher, at 2.1 ± 2.1%, than that by Choi’s in the group aged 20–39; however, it was lower by 5.6 ± 3.2% than that by Choi’s in the group aged 50 and above. Differences in the rate of decrease in pulmonary vital capacity with increasing age may arise from the ethnic characteristics of Koreans. In spite of the limited age group, a similar trend was shown with Lee’s equation; the average percentage of the FVC% by the NHANES III was higher in the group aged 40–49 than that by Lee’s equation, but lower in the group aged 50 and above.

In comparing concordance coefficients for the evaluation of the pulmonary dysfunction level between Choi’s and the non-Korean equations, differences were presented by age group; the concordance coefficient was lower in the group aged 50 and above than that in the group aged 20–39. However, for the concordance coefficient for the evaluation of restrictive pulmonary disease in the group aged 40–49, the agreement rate between Choi’s and the NHANES III equations was higher than that between the two Korean reference equations (Table 3, Table 6). This result could show the possibility that limited application of the NHANES III would be simple in the group aged 49 and below, that is, those who are comparatively young.

Among the five reference equations in this study, the spirometric interpretations and pulmonary dysfunction rates with Morris’s and Knudson’s equations were significantly different than those of the other three equations. It is difficult to conclude that these differences are caused by ethnic diversity because the NHANES III showed similar spirometric interpretations to those of the two Korean equations. Instead, these differences were likely caused by the differences in spirometric methods and devices used to develop Morris’s and Knudson’s equations due to their being developed longer ago. The Korean reference equations, including both Choi’s and Lee’s equations, may be free from method and device issues; however, their representativeness could be controversial because of the insufficient sample sizes used in their development. In spite of the limitation to comparing only the group aged 40 and above with the two Korean reference equations, spirometric interpretations from the two equations were close. Moreover, among the three non-Korean equations, the NHANES III showed the closest interpretations to those by the Korean reference equations although the result from the NHANES III did have some differences from the two Korean equations. Recently, in a US study based on the NHANES III equation, the estimation of spirometric reference values without ethnic categories has been suggested [22]. Considering this suggestion, ethnicity may not be a significant factor affecting spirometric reference values. Moreover, the similarity in interpretation from the NHANES III and the two Korean equations would be evidence for the validity of the Korean equations.

Thus, it can be concluded from the present study that the Korean equations are more reliable than Morris’s and Knudson’s equations. This study was performed based on occupational physical examinations in workers who had been exposed to pulmonary risk factors. In addition, the smoking rate of the subjects was higher than that of 39.6% in the general Korean male population [23].

Therefore, the exposure level for pulmonary risk factors in the subjects of this study was considered to be higher than that in the general population. The generalizability of the results of this study is limited to male workers with the ability to work. However, considering that in previous Korean studies, the subjects were male only, had respiratory disorders, and had an average age in the 60s, while in the present study, the average age was 44.5 and the subjects had the ability to work, which means the subjects were comparatively healthy, the results of this study should be closer than those of previous Korean studies to those expected in the general Korean population. According to the results of the present study, Morris’s and Knudson’s equations would be inappropriate to apply to spirometric screening for subjects with backgrounds similar to those of this study.

In this study, 80% of the spirometric reference value was defined as the minimum threshold of normal condition; however, the American Thoracic Society (ATS) has currently recommended that 95% of the spirometric reference value in a healthy population with no respiratory symptoms should be applied as the minimum threshold [19]. However, the threshold of 80% was considered to be appropriate for the definition of respiratory dysfunction in this study because the previous ATS guideline for impaired pulmonary function also used a threshold of 80% [24], and the legal value of 80% of the FVC and FEV_1_ has been widely used in Korea [25].

Up to the present, no particular spirometric reference equation has been recommended for the occupational physical examination in Korea. Caucasian reference equations such as Morris’s and Knudson’s equations, which are old versions, are more widely used than any Korean reference equations in Korea. One of the reasons for this could be that the Korean reference equations may not be well known. Another reason could be that institutions may have simply continued to use old equations based on existing policy. Finally, the Korean reference equations may not be included in the available spirometric equations programmed into locally used spirometric equipment. Thus, the Korean reference equations such as Choi’s equation may be difficult to apply for convenience’s sake. However, at minimum, consideration of a policy to abstain from applying Morris's and Knudson’s equations deserves the attention of both researchers and the Korean government.

## Conclusions

In this study, it was found that using Morris’s and Knudson’s spirometric reference equations should be avoided because their sensitivity for pulmonary dysfunction is too low in the Korean population. Applying the Korean reference equations is recommended for spirometry in the Korean population; however, the NHANES III and the Korean equations may complement each other because there is no significant difference between the two.

## Competing interests

This work was supported by grant from Inje university, 2010.

## Authors’ contributions

JT, CS, KH, CK and NK conceived of the study and participated its design. MH, NK and CS participated acquisition of data. KH, NK, SL, DH and SK performed the statistical analysis. NK drafted the manuscript. KH, CS, SK, JT, CK, SL and DH corrected and edited the manuscript. KH coordinated the study and helped to draft the manuscript. NK finalized the manuscript. All authors read and approved the final manuscript.
